# Some misunderstandings in psychology about confidence intervals

**DOI:** 10.3389/fpsyg.2022.948423

**Published:** 2022-07-22

**Authors:** Tadamasa Sawada, Lorick Huang, Oleg Y. Koryakov

**Affiliations:** ^1^School of Psychology, National Research University Higher School of Economics, Moscow, Russia; ^2^Akian College of Science and Engineering, American University of Armenia, Yerevan, Armenia; ^3^Department of Psychology, Russian-Armenian (Slavonic) University, Yerevan, Armenia; ^4^Institut Mathématiques de Toulouse, Toulouse, France

**Keywords:** confidence interval, normal distribution, T-distribution, quantitative literacy, quantitative psychology

## Introduction

Publication bias (e.g., Leggett et al., 2013; Francis, 2014) and the replication crisis (e.g., Open Science Collaboration, 2015) in empirical Psychological studies have been discussed especially intensively during the last 10 years (see Nelson et al., 2018 for a review). Many of these empirical studies report results that are too good to be true and the percentage of these studies in Psychology that can be successfully replicated has been estimated to be low.

These problems can be partly attributed to misunderstandings in Statistics. Cassidy et al. (2019) pointed out that the correct definition of a *p*-value is described only in 11% of Introduction-to-Psychology textbooks (see also Gigerenzer, 2018; Lakens, 2021). The assumptions underlying the central-limit theorem are often missing in the textbooks and the effectiveness of the central-limit theorem for securing normality of a distribution can be overestimated in Psychology (Hesterberg, 2008; Sawada, 2021). The standard error of the mean (SEM), instead of the standard deviation (SD), has been misused to describe variability across samples in Anesthesiology (Nagele, 2003). It seems likely that the same misuse can also be observed in Psychology.

**Figure 1:**
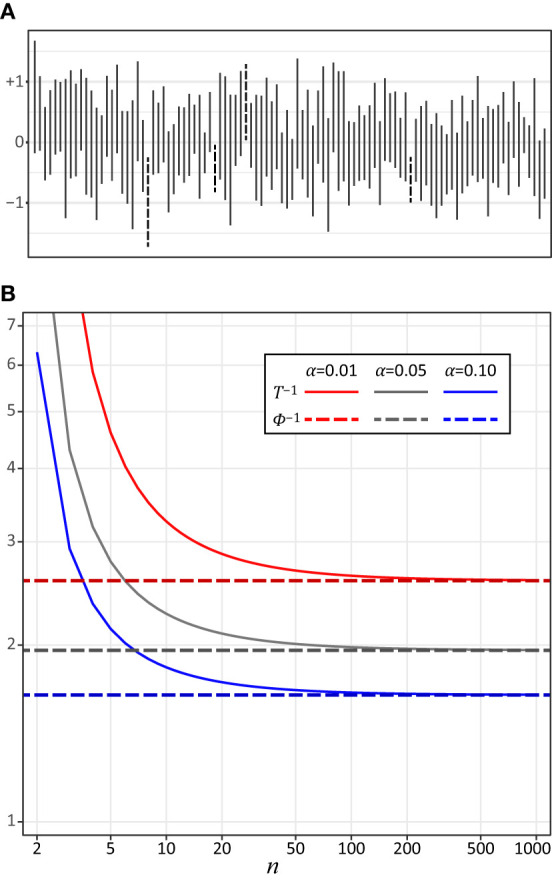
**(A)** The 95% CIs of the mean of the standard normal distribution computed from 100 sessions of a computer simulation. In each session of simulation, 10 samples were taken from a standard normal distribution and the CI was computed from samples based on a *t*-distribution (see Equation 1). The CIs that do not include the mean (0) are dotted. **(B)** The inverse cumulative distribution function *T*^−1^ of the *t*-distribution plotted as a function of the sample size *n*. The colors show the levels of α (α = 0.01, 0.05, and 0.10 for the 99, 95, and 90% CIs). The values of the inverse cumulative distribution function Φ^−1^ of the standard normal distribution are 2.58, 1.96, and 1.64 with α = 0.01, 0.05, and 0.10, respectively (shown in this graph as dashed lines).

Nowadays, authors of empirical Psychological studies are encouraged to report details of the Statistical tests and their results, including their confidence intervals (CI) when they report results of Psychological experiments (American Psychological Association, 2019). The CI of an unknown parameter is an interval estimate of the parameter. Consider repeatedly conducting a session during which you collect data and compute the CI of this unknown parameter with 100(1 – α)% (e.g., 95% when α = 0.05) of the confidence level. Note that the computed CIs change randomly across the sessions while the unknown parameter is regarded as a constant. With this done, a CI in each session will cover the parameter with a probability 100(1 – α)% (Figure 1A)^1^. So, 100(1 – α)% of the CIs estimated in the sessions include the unknown parameter asymptotically when the number of the sessions goes to positive infinity. The computation of the CI of an unknown parameter depends on the distribution used to characterize the relationship between the unknown parameter and its point estimate. Note that this distribution is used to test statistics of the parameter, e.g., a *t*-distribution of the mean of a normally-distributed population for both the *t*-test and for the CI of the mean (Devore, 2011). The CI represents the range of a value that is not significantly different from the point estimate of the unknown parameter with the 100α% level. The definition of the CI must be understood properly and it must be computed correctly if one is going to report the CI and to discuss empirical results based on it. Unfortunately, misunderstanding the CI is common (Hoekstra et al., 2014; Greenland et al., 2016).

In this study, we deliberately concentrated our discussion on the CI of the mean of the population because this is the most common CI used in Psychology, as represented in the Statistical textbooks that are currently being used in this field (these textbooks and their editions are listed in our Supplemental Material, https://osf.io/r7b8t/). We surveyed descriptions of the definition and of the equations used to compute the CI in the Statistics textbooks often used in Psychology. We found that the equation in 25% (5/20) of the textbooks computes the CI differently from the way the CI is computed in the other textbooks.

## The CI of the mean of a population

Consider a population that is normally distributed. The CI of the mean of this population is computed as:


(1) $$\left[\bar{\mu }-T^{-1}(1-\frac{\alpha }{2},\;n-1)\frac{\bar{\sigma }}{\sqrt{n}}\;\bar{\mu }+T^{-1}(1-\frac{\alpha }{2},\;n-1)\frac{\bar{\sigma }}{\sqrt{n}}\right]$$

where *n* is the sample size, $\bar{\mu }$ is the estimated mean of the population, $\bar{\sigma }$ is the estimated standard deviation and, $\frac{\bar{\sigma }}{\sqrt{n}}$ is the estimated standard error of the mean, and $T^{-1}\left(1-\frac{\alpha }{2},\;n-1\right)$ is the inverse cumulative distribution function of the *t*-distribution with the degree of freedom *n* – 1 and for the probability 1 – α/2. The level of the CI is 100(1 – α)% (e.g., α = 0.05 for the 95% CI). Note that the 95% CI of the mean is the range of the value from which the estimated mean $\bar{\mu }$ is *not* significantly different with the 100α% level (*p* ≥ α) when a single one-sample, two-tailed *t*-test is conducted^2^.

Note that the function $T^{-1}\left(1-\frac{\alpha }{2},\;n-1\right)$ can be well-approximated with the inverse cumulative distribution function of the standard normal distribution when the degree of freedom *n* – 1 is large enough (say *n* ≥ 30, see also Hesterberg, 2008). The inverse cumulative distribution function of the standard normal distribution is independent from *n*. If one assumes that the sample size *n* is large enough, the 100(1 – α)% CI can be written as:


(2) $$\left[\bar{\mu }-\Phi^{-1}(1-\frac{\alpha }{2})\frac{\bar{\sigma }}{\sqrt{n}}\bar{\mu }+\Phi^{-1}(1-\frac{\alpha }{2})\frac{\bar{\sigma }}{\sqrt{n}}\right]$$

where $\Phi^{-1}\left(1-\frac{\alpha }{2}\right)$ is the inverse cumulative distribution function of the standard normal distribution for the probability 1 – α/2. Note that $\Phi^{-1}\left(1-\frac{\alpha }{2}\right)$ is ~1.96 when α is 0.05. This number 1.96, alone, is occasionally introduced as a constant to compute the 95% CI in Psychology (e.g., Marks and Yardley, 2004).

The difference between Equations (1) and (2) is attributed to the distribution of the estimator (the estimated mean $\bar{\mu }$ of the population). Equation (1) is derived by using the *t*-distribution, while Equation (2) approximates the *t*-distribution with the standard normal distribution. This change of the distributions occurs because the standard deviation of the population is underestimated by the sample standard deviation $\bar{\sigma }$ computed from the samples when the sample size *n* is not sufficiently large. This bias of the sample standard deviation distorts the shape of the distribution of $\bar{\mu }$ from a normal distribution. If the sample size *n* is not sufficiently large, Equation (2) will underestimate the CI (Figure 1B)^3^. Both Equations (1) and (2) assume that the population is normally distributed in order to derive the distribution of $\bar{\mu }$. So, one must know the distribution of the estimator to compute the CIs.

Crucially, using Equation (2) instead of (1) means that the CI computed is asymptotic. In other words, it is only valid when the population is infinitely large. This means that the intuition we presented in the *Introduction* is no longer valid. When we repeat sessions, we have no guarantee that 100(1 – α)% of the intervals computed with equation (2) contain the true mean. A similar point can be made when the Central Limit Theorem (CLT) is used to circumvent the Gaussian assumption on the population.

Now, consider a population that is not normally distributed and its variance is a non-zero finite value. The CI of the mean of this population depends on the shape of the distribution of the population. According to the Central Limit Theorem, the CI can be computed by using Equation (2) asymptotically when the sample size goes to positive infinity (Dekking et al., 2005; Kwak and Kim, 2017). But, note that in a real experiment, the sample size will always be finite. The CI of the mean can be computed approximately by using Equation (1) when the sample size is finite and sufficiently large. Note that a “sufficiently-large” sample size depends on the shape of the distribution of the population (Cuadras, 2002; Wilcox, 2012). Mathematical bounds can be derived using Berry Essen's Theorem, and for some reason, the number 30 has become stuck in the literature (Hesterberg, 2008). Note, however, that more regularity through moment conditions is assumed, but hardly ever checked in practice.

If the population is not normally distributed and the sample size is small, the CIs computed using Equations (1) and (2) do not have any theoretically-valid meaning. For a non-normal distribution with a small sample size, the CI of the mean of the population is (Devore, 2011):


(3) $$\left[\bar{\mu }-\Pi_{\bar{\mu }-\mu }^{-1}(1-\frac{\alpha }{2})\bar{\mu }-\Pi_{\bar{\mu }-\mu }^{-1}(\frac{\alpha }{2})\right]$$

where μ is the true mean of the population and $\Pi_{\bar{\mu }-\mu }^{-1}$ is the inverse cumulative distribution function of the distribution of $\bar{\mu }-\mu$. Note that Equation (3) is theoretically valid but is not practically useful because the inverse cumulative distribution function $\Pi_{\bar{\mu }-\mu }^{-1}$ of $\bar{\mu }-\mu$ is generally unknown. There exists techniques for computing the CI from samples even when $\Pi_{\bar{\mu }-\mu }^{-1}$ is unknown (Rousselet et al., 2019) but the techniques relies on Bootstrap. These techniques need a sufficiently large sample size so that the empirical distribution is already close to the actual distribution of the population (Hesterberg, 2015).

We surveyed the equations used to compute the CI in the 20 Statistics textbooks often used in Psychology (these 20 textbooks and their editions are listed in our Supplemental Material, https://osf.io/r7b8t/). Five of the 20 textbooks were personally owned by the first author (TS). The other 15 textbooks were taken from the library at HSE University. Textbooks that do not describe any equation used to compute the CI were not included. We found that 75% (15/20) of the textbooks used Equation (1) that was based on a *t*-distribution to compute the CI. Ten of these 15 textbooks also described Equation (2) that is based on the normal distribution for a sample size that was sufficiently large. The remaining 25% (5/20) of the textbooks only used Equation (2).

## Discussion

In this study, we surveyed descriptions of the computation of the CI of the mean of a population in the 20 Statistics textbooks that are often used in Psychology today. The computation of the CI described in 75% (15/20) of the textbooks was based on a *t*-distribution (Equation 1) and the remaining 25% (5/20) of the textbooks used a different computation that was based on a normal distribution (Equation 2). The CIs computed by using these two different methods are different from one another. This inconsistency in the computation of the CI makes it difficult to compare results across studies quantitatively (e.g., meta-analysis).

Nowadays, psychologists are encouraged to report the results of their experiments with CIs (Wilkinson and Task Force on Statistical Inference American Psychological Association Science Directorate, 1999; Cumming and Finch, 2005). Considering the inconsistency across the textbooks that we noted, we encourage psychologists to describe how their CIs were computed (e.g., distributions, equations, or scripts) when they report CIs. Another remedy for this inconsistency is reporting estimated SDs or/and estimated SEMs with their sample sizes. Equations (1) and (2) show that the CI of the mean can be computed from the estimated SD or the estimated SEM when the sample size *n* is available. This means that the amount of information included in the CI that was computed from data is, theoretically, the same as the amount of data in the estimated SD as well as in the estimated SEM (see Francis, 2017). These inclusions can elucidate how the CIs were computed. Psychologists also need caution to read empirical studies reporting the CIs especially if the report does not have these inclusions.

It is worth pointing out that the CI in Psychology often refers to the CI of the mean of the population because only the CI of the mean of the population is described in many of the Statistics textbooks often used in Psychology. This reflects a current practice in Psychology that empirical data are discussed only on the basis of the difference of means across conditions. But, this is bad because it can discourage a quantitative discussion of other parameters (e.g., variance, median, and quantile) with their CIs. Note that the CIs of different parameters are computed by using different equations (see Harding et al., 2014 for a review).

Note that the issues of the CI that are discussed in this study are only a part of problems in the way that Psychologists handle statistics. Statistics education and the textbooks used in Psychology should be improved to address these problems.

## Author contributions

TS contributed to conception and design of the study. TS and OK conducted the survey. LH contributed the theoretical aspects of the study. TS and LH wrote the manuscript. All authors contributed to manuscript revision, read, and approved the submitted version.

## Conflict of interest

The authors declare that the research was conducted in the absence of any commercial or financial relationships that could be construed as a potential conflict of interest.

## Publisher's note

All claims expressed in this article are solely those of the authors and do not necessarily represent those of their affiliated organizations, or those of the publisher, the editors and the reviewers. Any product that may be evaluated in this article, or claim that may be made by its manufacturer, is not guaranteed or endorsed by the publisher.

## Footnotes

^1^The CI is often described as a range of values within which the unknown parameter falls with 100(1 – α)% of a probability. This description can lead to a misunderstanding, namely, that the unknown parameter is a random variable and that the CI of the parameter is fixed. This misunderstanding is especially problematical when multiple CIs of a single unknown parameter are discussed (e.g., a forest plot of a meta-analysis).

^2^Consider comparing the means of two populations using their CIs (Equation 1). Goodwin (2010) stated that, if the CI of the mean of one group does not overlap with the CI of the other group, the difference is necessarily significant (*p* < α) on the basis of an unpaired *t*-test (either equal or unequal variances). This statement is true but its inverse is not necessarily true. If the CIs of the means do overlap, the difference may or may not be significant (Schenker and Gentleman, 2001, see Cousineau et al., 2021 for adjusting the CI to compare the means of two populations).

^3^Consider computing two CIs using Equations (1) and (2) from 10 samples taken from a normal distribution. The CI computed by using Equation (1) is larger than the CI computed by using Equation (2) by a factor of 1.15 (=2.26/1.96). This smaller CI computed by using Equation (2) represents the range of a value that is not significantly different from the mean with the 8.2% level using a one-sample two-tailed *t*-test when the sample size is 10.
